# Antimicrobial Susceptibility Trends of Proteeae Isolates From a Tertiary-Care Hospital in Western Saudi Arabia

**DOI:** 10.7759/cureus.47494

**Published:** 2023-10-22

**Authors:** Rania A El-Kady, Samah A Alotaibi, Taef T Aljabri, Razan R Haraka, Imtinan M Ibrahim, Amal I Mousa, Mayar W Bashbeshi

**Affiliations:** 1 Medical Microbiology and Immunology, Faculty of Medicine, Mansoura University, Mansoura, EGY; 2 Pathological Sciences, Fakeeh College for Medical Sciences, Jeddah, SAU; 3 Medical School, Fakeeh College for Medical Sciences, Jeddah, SAU; 4 Medical Laboratory Sciences, Fakeeh College for Medical Sciences, Jeddah, SAU

**Keywords:** risk factors, multidrug-resistant, esbl, infection, proteeae

## Abstract

Background: The tribe *Proteeae* comprises *Proteus*, *Providencia*, and *Morganella* species. TheseGram-negative rods are of concern in that they are involved in diverse human infections, particularly in hospital settings. In the last two decades, there has been a sharp increase in infections by multidrug-resistant (MDR) *Proteeae*. Therefore, the objectives of this study were to: (i) assess the prevalence of infections caused by tribe *Proteeae*, (ii)determine the antimicrobial susceptibility profile of the test isolates, and (iii) identify the underlying risk factors for acquisition of infection by MDR strains.

Methods: During the period from January 2019 to December 2020, we conducted a retrospective review of the electronic medical and laboratory records of adult patients who received care at our institution. In addition, we analyzed the risk factors associated with acquisition of infections by members of the tribe *Proteeae* using univariate and multivariate regression models.

Results: Overall 403 adult patients (average age 59.69 ± 20.33 years) were enrolled into this study (196 males; 48.6%, and 207 females; 51.4%). *Proteus* *mirabilis* was the leading pathogen (70.7%; n=285), followed by *Morganella morganii *(20.1%; n=81), and* Providencia *species (9.2%; n=37). Most of the isolates were recovered from urine (59.3%; n=239), followed by wound swabs (23.1%; n=93), with the least from blood samples (1.7%; n=7). Out of 403 *Proteeae* isolates, 27.3% (n=110) were found to be extended-spectrum β-lactamase (ESBL)-producers, whereas 18.4% (n=74) were MDR. Patient’s age, concomitant diabetes mellitus (DM), and long hospital stays were independently associated with infection by MDR strains.

Conclusion: Infections by MDR *Proteeae* are leading causes for morbidity in our tertiary-care facility. Strict adherence to infection control precautions, as well as effective implementation of antimicrobial stewardship programs, are crucial to overcome these superbugs.

## Introduction

Members of the tribe *Proteeae *are ubiquitous in the environment and normal residents of the human gut microbiota. This tribe is composed of three genera, namely *Proteus*, *Morganella*, and *Providencia*. They are Gram‑negative, non-lactose fermenting rods that belong to the *Enterobacteriaceae*family of bacteria [1]. Interest in the tribe *Proteeae *has arisen mainly from a clinical perspective, as it includes a number of significant human pathogens that cause diverse community-acquired as well as healthcare-associated infections (HAIs) [2]. Contrary to *Escherichia coli*, *Proteeae *are more commonly recovered from patients with complicated urinary tract infections (UTIs) and are often associated with pyelonephritis, recurrent UTIs, and long duration of therapy [3]. Amongst them, *Proteus mirabilis *has been implicated in considerable and enduring infections in humans, including respiratory tract infections [4], wound infections, gastrointestinal, and UTIs [5]. 

The second member of the tribe *Proteeae*, *Morganella morganii*, was initially assumed to be an etiologic agent of summer diarrhea and was considered a negligible pathogen. However, in the early 1980s, Tucci and Isenberg described an outbreak of 13 *M*. *morganii *infections in a health-care facility. Since then, *M*. *morganii *has been recognized as a striking cause of HAIs [6]. Recently, a surge in infections caused by members of the genus *Providencia *has been reported. The most frequent species in the hospital milieu are *P*. *alcalifaciens*, *P*. *rettgeri*, *P*. *stuartii*, *P*. *rustigianii*, and *P*. *heimbachae*. Infections by this genus are increased in immunosuppressed patients, especially diabetics, and critically ill patients, and with long-term urinary catheterization [7].

The escalating trend of antimicrobial resistance is a global health threat. Members of the tribe *Proteeae *have intrinsic resistance to many classes of antibiotics, produce different types of β-lactamases, and show an intrinsic decreased sensitivity to imipenem, causing the treatment of these infections to be a therapeutic dilemma [8]. While some authors have described specific types of infections caused by these bacterial agents, limited studies have generally handled the *Proteeae *as a whole. With this in mind, we conducted this two-year retrospective study to: (i) determine the overall prevalence of infections by members of the tribe *Proteeae*, (ii) figure out the antimicrobial susceptibility pattern of the recovered isolates, and (iii) explore the underlying risk factors for acquisition of infections by multidrug-resistant (MDR) *Proteeae *from adult patients who attended the outpatient clinics or were admitted to Dr. Soliman Fakeeh Hospital (DSFH), Jeddah, Kingdom of Saudi Arabia (KSA).

## Materials and methods

Study design, subjects, and setting

In the period from January 2019 to December 2020, all adult patients (>18 years) who received care at DSFH were included in this retrospective cohort study if they had positive cultures for any of the organisms belonging to the tribe *Proteeae*. DSFH is a 500-bed tertiary-care institution in the Western region (Jeddah) of the KSA. It provides tertiary medical and surgical care for the residents of the Kingdom. 

Collection and identification of the bacterial isolates

Different clinical samples were collected from the study participants and processed in the Microbiology Laboratory of DSFH with reference to the standard protocols of the hospital laboratory. Bacterial isolates were identified based on their colony morphology, Gram staining characters, and results of biochemical tests. The VITEK 2 automated system (bioMérieux Brasil, Rio de Janeiro, Brazil) was used to accomplish the final identification processes.

Antibiotic susceptibility testing

The antibiotic susceptibility testing of the recovered isolates was investigated using the VITEK 2 system according to the manufacturer’s recommendations, using AST 291 cards. The following antibiotic disks were included in the testing panels: ampicillin (AMP), amoxicillin/clavulanate (AMC), piperacillin/tazobactam (TZP), ceftazidime (CAZ), ceftriaxone (CRO), cefepime (FEP), imipenem (IPM), meropenem (MEM), amikacin (AK), gentamicin (GN), tobramycin (TOB), ciprofloxacin (CIP), nitrofurantoin (F), trimethoprim/ sulfamethoxazole (SXT), and tigecycline (TGC). Interpretation of the susceptibility testing results was done according to the published guidelines of the Clinical and Laboratory Standards Institute (CLSI) [9,10]. The breakpoints established by the European Committee on Antimicrobial Susceptibility Testing (EUCAST) were applied to interpret tigecycline susceptibility results [11]. Production of extended-spectrum β-lactamases (ESBLs) in the test isolates was, also, determined using the VITEK 2 system. Isolates with MDR phenotypes (resistant to at least one agent in three or more antimicrobial categories) were identified with reference to the previously described definitions [12]. Quality control testing was performed using the CLSI-recommended quality control strains *Escherichia coli *ATCC 25922, *Klebsiella pneumoniae *ATCC 700603, *Pseudomonas aeruginosa* ATCC 27853, and *Proteus mirabilis *ATCC 35659.

Study participants’ data extraction

During the period of interest, we reviewed the hospital electronic medical records of the enrolled patients using the medical record numbers (MRN) to extract the relevant data to the study cohort, including; patients’ demographics, comorbidities, invasive procedures, surgical intervention, collected samples, administered antibiotics, intensive care unit (ICU) admission, and length of hospital stay (LOS). Furthermore, we analyzed the electronic database of the Microbiology Laboratory of DSFH to capture the data related to the recovered microorganisms and their antimicrobial susceptibility patterns.

Exclusion criteria

Pediatric patients (<18 years), patients with multiple sites of infection or polymicrobial infections, and patients with missing clinical data or lacking susceptibility test profiles were excluded from the study cohort.

Ethical considerations

The current study was performed in keeping with the ethical principles of the Declaration of Helsinki. The study protocol was granted approval by the institutional review board (IRB) of DSFH (Decision no. 191/IRB/2021), and the need for informed consent was waived since all data were anonymized before analysis. Patients’ data privacy and confidentiality were respected in all phases of the study.

Statistical analysis

All data were analyzed using SPSS® Statistics program version 26.0 for Windows (IBM Corp., Armonk, NY, USA). Continuous variables were described as means ± standard deviation (SD) for parametric data after testing normality using the Kolmogrov-Smirnov test. The independent samples *t*-tests were used to analyze the means of two independent groups. Categorical variables were described as numbers and percentages with Pearson’s Chi-Square (*χ*^2^) test used for comparison. Odds ratios (ORs) and 95% confidence intervals (CIs) were determined to evaluate the strength of any association. Univariate and multivariate logistic regression analyses were done to detect the risk factors for acquisition of infection by MDR *Proteeae *isolates. The difference was considered statistically significant at P-values ≤ 0.05 (2-tailed).

## Results

Demographics and baseline features of the study cohort

The present study included 403 patients (196 males; 48.6%, and 207 females; 51.4%), with an average age of 59.69 ± 20.33 years (range 18-99 years). About 29% (n=118) of the study cohort were diabetics, whereas 16.8% (n=68) suffered from concomitant chronic kidney diseases (CKD). The mean LOS was 26.57 ± 29.19 days (range 4-133 days). Amongst our participants, 19.4% (n=78) were admitted to the ICU, while 8.2% (n=33) underwent surgical intervention. About 48% of our patients (n=192) received antibiotics in the last month prior to acquisition of infection.

Distribution of the study isolates

During the period of interest, a total of 403 non-duplicate (one isolate/patient), consecutive isolates belonging to the tribe *Proteeae *were identified, of which *P*. *mirabilis *(70.7%; n=285) was the most common pathogen, followed by *M*. *morganii* (20.1%; n=81), and *Providencia* species (7.5% *P*. *stuartii*; n=30, and 1.7% *P*. *rettgeri*; n=7). Distribution of the identified isolates per sample is shown in Table 1. The majority of the isolates were recovered from urine (59.3%; n=239), followed by wound swabs (23.1%; n=93) and the least from blood samples (1.7%; n=7).

**Table 1:** Sample-wise distribution of the recovered Proteeae isolates Data are presented as numbers (n) and percentages (%)

Samples	Proteus mirabilis		Morganella morganii		Providencia stuartii		Providencia rettgeri		Total	
	n	%	n	%	n	%	n	%	n	%
Urine	169	70.7	54	22.6	13	5.4	3	1.3	239	100
Wound swabs	65	69.9	14	15.1	11	11.8	3	3.2	93	100
Body fluids	19	63.3	10	33.3	1	3.4	0	0.0	30	100
Pus	16	84.2	2	10.5	1	5.3	0	0.0	19	100
Sputum	10	66.7	1	6.7	3	20.0	1	6.7	15	100
Blood	6	85.7	0	0.0	1	14.3	0	0.0	7	100

Antimicrobial resistance patterns of the recovered *Proteeae *isolates

Overall, high resistance rates to nitrofurantoin (93.5%; n=158 out of 169), ampicillin (74.7%; n=213 out of 285), and amoxicillin-clavulanate (54.7%; n=156 out of 285) were observed amongst *P*. *mirabilis *isolates, whereas the least resistance was to meropenem (16.8%; n=48). Of 403 *Proteeae *isolates, 27.3% (n=110) and 18.4% (n=74) were found to be ESBL-producers and MDR, respectively. The detailed antibiotic testing results of all *Proteeae *isolates are depicted in Table 2.

**Table 2:** Antimicrobial resistance profiles of the test Proteeae isolates AMP, ampicillin; AMC, amoxicillin-clavulanate; TPZ, piperacillin/tazobactam; CAZ, ceftazidime; CRO, ceftriaxone; FEP, cefepime; AK, amikacin; GN, gentamicin; TOB, tobramycin; CIP, ciprofloxacin; F, nitrofurantoin; SXT, trimethoprim/ sulfamethoxazole; IPM, imipenem; MEM, meropenem; TGC, tigecycline; ESBL, extended-spectrum β-lactamase; MDR, multidrug-resistant; NT, not tested Tobramycin (TOB) was tested only against isolates recovered from wound swabs and nitrofurantoin (F) was tested only against urinary isolates. Data are expressed as numbers (n) and percentages (%)

Antibiotics	Proteus mirabilis		Morganella morganii		*Providencia *species	
	n=285	%	n=81	%	n=37	%
AMP	213	74.7	NT	–	NT	–
AMC	156	54.7	NT	–	NT	–
TZP	69	24.2	19	23.5	7	18.9
CAZ	110	38.6	26	32.1	17	45.9
CRO	102	35.8	23	28.4	14	37.8
FEP	77	27.0	12	14.8	12	32.4
GN	81	28.4	21	25.9	10	27
AK	30	10.5	4	4.9	2	5.4
TOB	20/65	30.8	3/14	21.4	6/14	42.9
CIP	83	29.1	17	21	6	16.2
F	158/169	93.5	50/54	92.6	14/16	87.5
SXT	155	54.4	30	37	13	35.1
IPM	57	20.0	18	22.2	7	18.9
MEM	48	16.8	9	11.1	4	10.8
TGC	NT	–	40	49.4	14	37.8
ESBL-producers	89	31.2	13	16	8	21.6
MDR isolates	59	20.7	10	12.3	5	13.5

Sources for multidrug-resistant *Proteeae *isolates

Out of 74 identified MDR *Proteeae *isolates, 42 (56.7%) were recovered from urine samples, 19 (25.7%) from wound swabs, and four (5.4%) from pus samples. In addition, four (5.4%), three (4.1%), and two (2.7%) of the MDR isolates were obtained from body fluids, blood, and sputum samples, respectively.

Risk factors for infection by multidrug-resistant *Proteeae *isolates

In the univariate analysis, patient age, diabetes mellitus (DM), CKD, and concomitant pulmonary disease were identified as predisposing factors for infection with MDR *Proteeae*. In addition, insertion of central venous catheter (CVC) or indwelling urinary catheter (IUC), prior exposure to antibiotics, ICU admission, and lengthy hospital stays were associated with increased risk of infection with MDR *Proteeae *as shown in Table 3.

**Table 3:** Risk factors associated with acquisition of infection by multidrug-resistant Proteeae isolates MDR, multidrug-resistant; χ2, Pearson’s Chi-Square; SD, standard deviation; DM, diabetes mellitus; CKD, chronic kidney disease; CHD, chronic heart disease; CLD, chronic liver disease; PVC, peripheral venous catheter; CVC, central venous catheter; IUC, indwelling urinary catheter; MV, mechanical ventilation; ICU, intensive care unit; LOS, length of hospital stay Data are expressed as numbers and percentages unless otherwise indicated; a: Significance was tested using the independent samples *t*-tests; *P < 0.05 (statistically significant)

Risk factors	MDR *Proteeae *isolates (n=74)	Non-MDR *Proteeae *isolates (n=329)	*χ*^2^	*P*-value
Age, years (± SD)^a^	76.35 ± 14.59	55.94 ± 19.56	8.46	< 0.0001*
Gender				
Males Females	38 (51.4%) 36 (48.6%)	158 (48%) 171 (52%)	0.27	0.61
Underlying morbidity				
DM	43 (58.1%)	75 (22.8%)	36.38	< 0.0001*
CKD	25 (33.8%)	43 (13.1%)	18.48	< 0.0001*
CHD	11 (14.9%)	44 (13.4%)	0.11	0.741
CLD	8 (10.8%)	41 (12.5%)	0.15	0.687
Malignancy	6 (8.1%)	16 (4.9%)	1.23	0.267
Lung disease	15 (20.3%)	10 (3%)	30.83	< 0.0001*
Invasive procedures				
PVC	25 (33.8%)	78 (23.7%)	3.22	0.073
CVC	17 (23%)	39 (11.9%)	6.24	0.012*
IUC	19 (25.7%)	39 (11.9%)	9.37	0.002*
MV	8 (10.8%)	21 (6.4%)	1.77	0.183
Surgery	4 (5.4%)	29 (8.8%)	0.93	0.334
Prior antibiotics	46 (62.2%)	146 (44.4%)	7.66	0.006*
ICU admission	30 (40.5%)	48 (14.6%)	26.07	< 0.0001*
LOS, days (± SD)^a^	72.14 ± 30.16	16.33 ± 16.39	22.09	< 0.0001*

Independent predictors for acquisition of infection by multidrug-resistant *Proteeae *isolates

In the multivariate logistic regression model, old age, DM, and increased LOS were identified as independent predictors for infection by MDR *Proteeae *as given in Table 4.

**Table 4:** Independent predictors for acquisition of infection by multidrug-resistant Proteeae isolates MDR, multidrug-resistant; DM, diabetes mellitus; CKD, chronic kidney disease; CVC, central venous catheter; IUC, indwelling urinary catheter; ICU, intensive care unit; LOS, length of hospital stay; OR, odds ratio; CI, confidence interval Data are expressed as numbers; *P < 0.05 (statistically significant)

Risk factors	Univariate analysis			Multivariate analysis		
	OR	95% CI	*P-*value	OR	95% CI	*P-*value
Age	1.78	1.16–2.74	< 0.0001	0.93	0.90–0.96	< 0.0001*
DM	0.21	0.13–0.36	< 0.0001	4.35	1.63–11.66	0.003*
CKD	0.29	0.17–0.53	< 0.0001	0.58	0.16–2.05	0.39
Lung diseases	0.12	0.05–0.29	< 0.0001	4.66	0.93–23.39	0.06
CVC	0.45	0.24–0.85	0.012	0.54	0.08–3.72	0.53
IUC	0.39	0.21–0.72	0.002	1.31	0.22–7.88	0.76
Prior antibiotics	0.48	0.28–0.82	0.006	1.03	0.42–2.53	0.94
ICU admission	0.25	0.14–0.44	< 0.0001	1.29	0.31–5.31	0.71
LOS	4.77	3.11–7.17	< 0.0001	0.93	0.91–0.95	< 0.0001*

## Discussion

Infections attributable to the tribe *Proteeae *are cosmopolitan and were formerly controllable using the available antibiotics. However, rates of antibiotic resistance in these organisms have been dramatically increased, especially in healthcare settings. In this context, our study deemed to identify the frequency of *Proteeae *isolates, their antimicrobial resistance profiles, and the risk factors associated with the emergence of MDR strains in our institution.

During the study period, 403 non-repetitive isolates belonging to the *Proteeae *were collected from the study cohort, accounting for 6.1% of the total isolates (n=6,558), compatible with the data from a previous prospective study carried out in a Romanian intensive care unit [13]. This finding denotes that these microorganisms are rather frequent in our institution, which advocates periodic surveillance and monitoring to establish their associated epidemiology. Of these isolates, *P*. *mirabilis *was the leading organism (70.7%; n=285), followed by *M*. *morganii *(20.1%; n=81) and *Providencia *species (9.2%; n=37), in keeping with the results from a previous Canadian population-based laboratory surveillance [14]. In an earlier study, in a tertiary-care facility in Korea involving 132 cases with bacteraemia, *M*. *morganii *was the most common isolate followed by *Proteus *species [15]. The somewhat different distribution could be ascribed to diverse geographic regions and different infection control strategies in various healthcare facilities.

In our study, sample-wise distribution of the collected *Proteeae *isolates showed the highest frequency from urine samples (59.3%; n=239) followed by wound swabs (23.1%; n=93), whereas the lowest rate was identified from blood samples (1.7%; n=7). This finding aligns with the results from a previous study, in which most of *Proteeae *isolates were recovered from urine samples followed by wound swabs [14], highlighting the clinical relevance of *Proteeae *as causative agents for UTIs. Opposed to UTIs caused by other bacterial agents, UTIs caused by *Proteeae *are more severe and have increased risk of recurrence, complications, and pyelonephritis [16]. 

Treatment of *Proteeae *infections is particularly burdensome, owing to the multiple intrinsic resistance mechanisms they harbor. Current evidence has shown that the last-choice antibiotics which are useful in difficult-to-treat infections by Gram-negative bacteria, including colistin, are not useful in treatment of *Proteeae *infections [8]. In the existing study, high rates of resistance to nitrofurantoin, ampicillin, and amoxicillin-clavulanate were observed amongst *P*. *mirabilis *isolates (93.5%, 74.7% and 54.7%, respectively), similar to the rates reported from Jordan [17]. This finding could be related to the indiscriminate use of these antibiotics for treatment of community-acquired UTIs. In addition, 29.1% (n=83) and 28.4% (n=81) of *P*. *mirabilis *isolates were resistant to ciprofloxacin and gentamicin, respectively, in line with the published data from elsewhere [18]. Our local resistance pattern reflects a growing tendency of *P*. *mirabilis *towards resistance to a multitude of antibiotics. Also, it emphasizes the need for regular surveillance so as to figure out treatment guidelines for local use. On the other side, a 10.5% resistance rate was observed to amikacin, 16.8% to meropenem, and 20% to imipenem, signifying that these antimicrobials can be deployed for treatment of *P*. *mirabilis *infections. 

In the present study, 27.3% of the *Proteeae *isolates (n=110) were found to be ESBL producers, with the highest rate detected among *P*. *mirabilis *(31.2%; n=89). The increased rates of ESBL production amid our isolates could be attributed to the selective pressure generated by the widespread use of third-generation cephalosporins in our institution, associated with lack of routine molecular characterization of the recovered isolates. Our findings are fairly parallel to the results from a previous Nigerian study, where 21% of the investigated *Proteeae *were confirmed phenotypically to be ESBL producers [19]. Amongst our *P*. *mirabilis *isolates, the rate of ESBL production is considerably lower than that from a recent report from Egypt [20]. On the other side, 21.6% of the recovered *Providencia *species isolates (n=8) produced ESBLs, compared to 52% from an Italian university hospital [21]. These variations could be allocated to regional dissimilarity in bacterial strains, prevalence of virulence genes, and different degrees of adherence to infection control practices.

Over recent years, some reports on the development and propagation of MDR *Proteeae *have been published. Our study demonstrated that 18.4% of the recovered *Proteeae *were MDR (n=74), which is lower than the findings from a University Hospital in Western Romania, where 44.5% of *Proteeae *isolates exhibited MDR phenotypes [22]. Amongst the test isolates, *P*. *mirabilis *showed the highest MDR rate (20.7%). Almost half of the MDR *P*. *mirabilis *isolates (50.8%; 30/59) were obtained from urine samples. This result specifies that *P*. *mirabilis *has a greater predisposition for colonizing the urinary tract and exerting resistance to various antibiotics [20]. A recent single-centered, observational, retrospective study from Saudi Arabia reported a lower rate of MDR amongst *Proteus *species (12.5%). This discrepancy could be ascribed to different study design, where only patients diagnosed with UTIs were included in that study [23].

At present, *M*. *morganii *is considered an emerging clinical treatment challenge, secondary to its intrinsic resistance genes. Also, the enduring acquisition of resistance genes or virulence factors puts this organism on the track of being the future superbug. In a recent retrospective, population-based surveillance for *M*. *morganii *bloodstream infections (BSIs) from Australia, MDR strains accounted for 9.5% compared to 6.3% of our *M*. *morganii *isolates [24]. In a contemporary study conducted at the United States Medical Centers (2018-2022), 9.8% of *P*. *stuartii *isolates had an MDR profile compared to 13.5% in the current study [25]. Data from another Korean study described an upsetting outbreak of eight urinary isolates of MDR *P*. *rettgeri *at a surgical ICU, through which all of the isolates were resistant to cephalosporins, aztreonam, meropenem, ertapenem, ciprofloxacin, and amikacin. The outbreak continued for 13 months, and was finally eliminated after reinforcement of infection control measures in the involved ICU [26]. So, efforts to promote infection control practices in healthcare centers are mandatory.

In an attempt to tackle the spread of MDR pathogens, risk factors for acquisition of infection need to be identified to plan powerful methods for prevention and control. Amongst our cohort, old age, being diabetic, and longer LOS were independently associated with acquisition of infections by MDR *Proteeae *isolates. Evidence from other studies indicates that elderly patients are more susceptible to infections, mostly due to their compromised immune system and sequelae of chronic diseases [27]. In our study, diabetic patients were four times more likely to develop infections with MDR *Proteeae *(OR=4.35, 95% CI: 1.63-11.66; P=0.003), which coincides with the observations of Rus et al. [28]. Jeopardized host immune response along with reduced inflammatory cells may be leading factors to MDR bacterial infections in diabetic patients. 

Intriguingly, Abdallah et al. (2018) described the first case of carbapenem-resistant *P*. *stuartii *in a 31-year-old man who contracted hospital-acquired pneumonia (HAP) in an ICU in Riyadh, Saudi Arabia. The widespread use of colistin, tigecycline and carbapenems in the ICU, receipt of multiple courses of antibiotics, and prolonged hospital stay may be contributing factors for the emergence of this mutant strain in the patient, that responded to a two-week regimen of double-dose meropenem provided as an extended infusion over three hours [29]. Furthermore, statistics from another Indian study revealed that prior antibiotic administration and use of urinary catheters are major drivers for infection with MDR strains, corresponding to our findings [30]. So, endorsing antibiotic stewardship, optimizing the use of invasive devices, and minimizing unnecessary hospital days are key pillars to prevent infections with MDR *Proteeae*.

Our study has some limitations that are worth mentioning. Firstly, the retrospective design makes it liable to selection bias. Secondly, it is a unicentric study conducted at a single tertiary hospital, which may limit globalization of our findings to other regions of the kingdom. Lastly, the underlying molecular mechanisms for the emergence of resistant traits were not deciphered.

## Conclusions

Antimicrobial resistance is a growing concern worldwide, and is the foremost barrier to eradicating bacterial infections. In our study, MDR *Proteeae* accounted for 18.4% of the recovered *Proteeae *isolates. Thereby, close vigilance of the incidence and spread of resistant strains is critical due to the negative impact they pose on the current antimicrobial armamentarium. What's more, our findings underscore the urgent need for promoting antibiotic stewardship programs and infection control strategies in our healthcare center to curb further spread of resistant strains in the healthcare settings and subsequent spill-over into the community.
